# Discrete-State Stochastic Models of Calcium-Regulated Calcium Influx and Subspace Dynamics Are Not Well-Approximated by ODEs That Neglect Concentration Fluctuations

**DOI:** 10.1155/2012/897371

**Published:** 2012-12-29

**Authors:** Seth H. Weinberg, Gregory D. Smith

**Affiliations:** Department of Applied Science, The College of William and Mary, Williamsburg, VA 23187, USA

## Abstract

Cardiac myocyte calcium signaling is often modeled using deterministic ordinary differential equations (ODEs) and mass-action kinetics. However, spatially restricted “domains” associated with calcium influx are small enough (e.g., 10^−17^ liters) that local signaling may involve 1–100 calcium ions. Is it appropriate to model the dynamics of subspace calcium using deterministic ODEs or, alternatively, do we require stochastic descriptions that account for the fundamentally discrete nature of these local calcium signals? To address this question, we constructed a minimal Markov model of a calcium-regulated calcium channel and associated subspace. We compared the expected value of fluctuating subspace calcium concentration (a result that accounts for the small subspace volume) with the corresponding deterministic model (an approximation that assumes large system size). When subspace calcium did not regulate calcium influx, the deterministic and stochastic descriptions agreed. However, when calcium binding altered channel activity in the model, the continuous deterministic description often deviated significantly from the discrete stochastic model, unless the subspace volume is unrealistically large and/or the kinetics of the calcium binding are sufficiently fast. This principle was also demonstrated using a physiologically realistic model of calmodulin regulation of L-type calcium channels introduced by Yue and coworkers.

## 1. Introduction

Concentration changes of physiological ions and other chemical species (such as kinases, phosphatases, and various modulators of cellular activity) influence and regulate cellular responses [1]. These dynamics are often modeled using systems of deterministic ordinary differential equations (ODEs) that assume chemical species concentrations are nonnegative real-valued quantities (i.e., the state-space is continuous). In such descriptions, the rate of change of the concentration of each species is usually specified under the assumption of mass-action kinetics, that is, the rate of a reaction is proportional to the product of reactant concentrations. However, under physiological conditions the concentrations of chemical species are often quite low and, in some cases, restricted subspaces in which these species are contained are very small. For example, L-type calcium channels in cardiac myocytes are typically clustered in small “diadic subspaces” that have a volume of ~10^−17^ liters, with approximately 20,000 diadic subspaces per cell [2, 3]. Resting calcium concentration in the diad is typically 0.1 micromolar, a value that corresponds to an average of 0.6 calcium ions per subspace [4]. Because only whole numbers of calcium ions can be present in a subspace at any given time, the question arises: *is it appropriate to use deterministic ODEs to model subspace calcium dynamics?*

Previous studies have compared discrete-state (stochastic) and continuous-state (deterministic) models in the analysis of biological and chemical systems, including models of biochemical networks, enzyme kinetics, and population dynamics [5–21]. These studies have shown that in the “large-system limit" (i.e., a large “copy number” of each chemical species), the solution of discrete and continuous models are equivalent [12]. However, for a small system, concentration values obtained from a continuous deterministic model (an approximation that neglects concentration fluctuations) can significantly deviate from the expected value obtained from the discrete stochastic model. When chemical reactions are higher than first order, there is no guarantee that the deterministic mass-action formulation will agree with, or be a good approximation to, the expected value of species concentrations obtained from a chemical master equation that accounts for discrete system states and concentration fluctuations [5]. An excellent study by Goutsias discusses the relationship between the discrete and continuous formulations for general biochemical systems [22] (for theoretical context, see [23]).

Because of recent interest in the physiological relevance of spatially localized control of voltage- and calcium-regulated calcium influx and sarcoplasmic reticulum calcium release in cardiac myocytes [24–26], we sought to determine precisely when the conventional deterministic formulation of these processes are a valid approximation. When is it appropriate to model the dynamics of subspace calcium using deterministic ODEs? When does one require a stochastic description that accounts for the fundamentally discrete nature of calcium-regulated calcium influx?

To answer this question, we constructed and analyzed a minimal Markov model of a calcium-regulated calcium channel and associated subspace. We compared the expected steady-state subspace calcium concentration in this stochastic model (a result that accounts for the small subspace volume) with the result obtained using the corresponding deterministic ODE model (an approximation that assumes large system size).  Section 2.1 introduces our model formulation and shows the agreement between deterministic and stochastic descriptions when subspace calcium does not regulate calcium influx. However, when calcium binding regulates channel activity (through either activation or inactivation), the deterministic and stochastic descriptions often disagree (Sections 2.2 and 2.3). In general, the effect of concentration fluctuations in a spatially restricted calcium domain with a calcium-regulated calcium influx pathway (e.g., a stochastically gating L-type calcium channel) is only well-approximated by the deterministic description when the subspace volume is sufficiently (unphysiologically) large or the kinetics of calcium binding to the calcium-regulated channel are sufficiently fast. This principle was also demonstrated using a physiologically realistic model of calmodulin regulation of L-type calcium channels produced by Yue and coworkers (Section 2.4).

## 2. Methods and Results

### 2.1. Calcium Influx and Subspace Calcium Concentration Fluctuations

**Figure 1:**
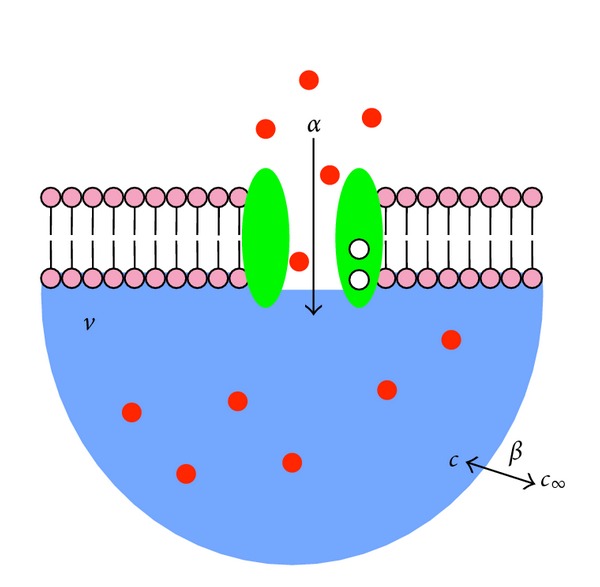
Diagram of the components and fluxes in a minimal subspace model. Calcium influx *α* (in units of *μ*M/s) leads to increased calcium concentration *c* (units of *μ*M) in a diadic subspace of volume *v* (liters). Subspace calcium moves to the bulk passively via diffusion at rate *β* (given by 0.01 ms^−1^). Bulk calcium at the concentration *c*_*∞*_ = 0.1 *μ*M returns to the subspace at the same rate. The equilibration time of subspace calcium is *τ* = 1/*β* = 100 ms [27].

 We begin with the case of a single calcium channel that is associated with a spatially restricted subspace but not regulated by subspace calcium (Figure 1 and Section 2.1). The description of the model in the absence of calcium regulation simplifies the initial presentation of the model and allows us to illustrate general properties of subspace calcium concentration fluctuations. Subsequently, we present a more complete model formulation that includes calcium-regulated calcium influx (Figure 3 and Section 2.2). For simplicity, we neglect the presence of endogenous calcium binding proteins and assume a constant flux of calcium, denoted by *α*, into the subspace. The subspace calcium concentration is passively coupled with relaxation rate *β* = 0.01 ms^−1^ to the constant bulk concentration of *c*_*∞*_ = 0.1 *μ*M [27]. These assumptions lead to the following deterministic model of subspace calcium dynamics:

(1) $$\begin{array}{c} \frac{dc}{dt}=\alpha -\beta \left(c-c_{\infty }\right), \end{array}$$

where the influx rate *α* has units of concentration per time (e.g., *μ*M/ms) and the calcium concentration *c* is a continuous real-valued quantity.

#### 2.1.1. Stochastic Model

In the corresponding stochastic description of calcium influx into a diadic subspace, the state variable is the number of calcium ions in the subspace (a discrete quantity that we will denote by $\hat{\text{C}}\in \{0,1,\ldots ,\infty \}$, where the caret (hat) indicates that $\hat{\text{C}}$ is a dimensionless number of molecules rather than concentration, and the capitalization indicates a random variable. The fluctuating subspace calcium concentration (also a random variable, denoted by C) depends on both $\hat{\text{C}}$ and the subspace volume (*v*), that is,

(2) $$\begin{array}{c} \text{C}=\frac{\hat{\text{C}}}{v}. \end{array}$$

Using this relationship, it is straightforward to derive the transition rates between the discrete states of the stochastic model that are consistent with (1). The resulting state-transition diagram for the stochastic model is

(3) $$\begin{array}{c} 0\overset{\overset{-}{\alpha }}{\underset{\beta }{⇌}}1\overset{\overset{-}{\alpha }}{\underset{2\beta }{⇌}}2\cdots n-1\overset{\overset{-}{\alpha }}{\underset{n\beta }{⇌}}n\overset{\overset{-}{\alpha }}{\underset{\left(n+1\right)\beta }{⇌}}n+1\cdots , \end{array}$$

where the index that labels states, *n* ∈ {0,1,…, *∞*}, ranges over all possible numbers of calcium ions in the subspace and the constant $\overset{-}{\alpha }$ is proportional to the subspace volume, that is,

(4) $$\begin{array}{c} \overset{-}{\alpha }=v\left(\alpha +\beta c_{\infty }\right). \end{array}$$

#### 2.1.2. Master Equation and Steady-State Probability Distribution

If we write $p_{n}(t)=\text{Pr}\{\hat{\text{C}}(t)=n\}$, the equations for the dynamics of the probability of each state in (3), that is, the chemical master equation for the number of calcium ions in the subspace, is given by

(5) $$\begin{array}{c} \frac{dp_{0}}{dt}=-\overset{-}{\alpha }p_{0}+\beta p_{1},\\ \frac{dp_{n}}{dt}=-\left(\overset{-}{\alpha }+n\beta \right)p_{n}+\overset{-}{\alpha }p_{n-1}+\left(n+1\right)\beta p_{n+1},\;n=1,2,\ldots . \end{array}$$

Note that the correspondence between the rate constants in the deterministic (1) and stochastic (3)–(5) models is established by substituting $c=\hat{c}/v$ in (1) to find the rate of change of the number of calcium ions in the deterministic model, that is,

(6) $$\begin{array}{c} \frac{d\hat{c}}{dt}=\overset{-}{\alpha }-\beta \hat{c}. \end{array}$$

This equation indicates that $\hat{c}$ increases at rate $\overset{-}{\alpha }$ (due to influx and diffusion from the bulk), a value that is independent of the number of calcium ions in the subspace. At the same time, $\hat{c}$ decreases at rate $\beta \hat{c}$, a value that is proportional to $\hat{c}$ because each ion has an opportunity to diffuse into the bulk. Consequently, the transition rates leading out of state $\hat{\text{C}}=n$ in the stochastic model are given by $\overset{-}{\alpha }$ for the $\hat{\text{C}}=n$ to *n* + 1 transitions and *βn* for the $\hat{\text{C}}=n$ to *n* − 1 transitions.

To find the steady-state probability distribution of $\hat{\text{C}}$, we set the left hand sides of (5) to zero to obtain

(7) $$\begin{array}{c} n\beta p_{n}=\overset{-}{\alpha }p_{n-1},\;n=1,2,\ldots \end{array}$$

from which it follows that {*p*_*n*_} is a Poisson distribution with parameter $\lambda =\overset{-}{\alpha }/\beta$, that is,

(8) $$\begin{array}{c} p_{n}=e^{-\lambda }\frac{\lambda^{n}}{n!}. \end{array}$$

#### 2.1.3. Analysis of Concentration Fluctuations

To see how the subspace calcium concentration fluctuations predicted by this minimal model depend on the parameters *α*, *β*, *c*_*∞*_, and *v*, recall that the mean and variance of the Poisson distribution (8) is equal to the parameter *λ* and, consequently, the steady-state expected number of calcium ions in the subspace is given by

(9) $$\begin{array}{c} \text{E}\left[\hat{\text{C}}\right]=\sum_{n=0}^{\infty }np_{n}=\lambda =\frac{\overset{-}{\alpha }}{\beta }=v\left(\frac{\alpha }{\beta }+c_{\infty }\right)={vc}_{*}, \end{array}$$

where the last equality defines *c*_∗_ as follows:

(10) $$\begin{array}{c} c_{*}=\frac{\alpha }{\beta }+c_{\infty }. \end{array}$$

Using (9) and the fact that $\text{C}=\hat{\text{C}}/v$ implies $\text{E}[\text{C}]=\text{E}[\hat{\text{C}}]/v$, we can identify *c*_∗_ as the expected subspace calcium concentration:

(11) $$\begin{array}{c} \text{E}\left[\text{C}\right]=c_{*}. \end{array}$$

Similarly, the steady-state variance of the number of calcium ions in the subspace is

(12) $$\begin{array}{c} \mathrm{Var}\left[\hat{\text{C}}\right]=\sum_{n=0}^{\infty }{\left(n-\text{E}\left[\text{C}\right]\right)}^{2}p_{n}={vc}_{*}, \end{array}$$

and $\mathrm{Var}[\text{C}]=\mathrm{Var}[\hat{\text{C}}]/v^{2}$ implies that the variance of the subspace calcium concentration is

(13) $$\begin{array}{c} \mathrm{Var}\left[\text{C}\right]=\frac{c_{*}}{v}. \end{array}$$

Note that the coefficient of variation of $\hat{\text{C}}$ and C are identical and inversely proportional to subspace volume, that is, $\text{CV}[\hat{\text{C}}]={\left(\mathrm{Var}[\hat{\text{C}}]\right)}^{1/2}/\text{E}[\hat{\text{C}}]=1/\sqrt{vc_{*}}$ and, similarly,

(14) $$\begin{array}{c} \text{CV}\left[\text{C}\right]=\frac{\sqrt{\mathrm{Var}\left[\text{C}\right]}}{\text{E}\left[\text{C}\right]}=\frac{\sqrt{\mathrm{Var}\left[\hat{\text{C}}\right]/v^{2}}}{\text{E}\left[\hat{\text{C}}\right]/v}=\frac{1}{\sqrt{vc_{*}}}. \end{array}$$

This is a well-known principle from statistical physics: fluctuation amplitudes scale with the reciprocal of the square root of system size (the subspace volume *v*).

**Figure 2:**
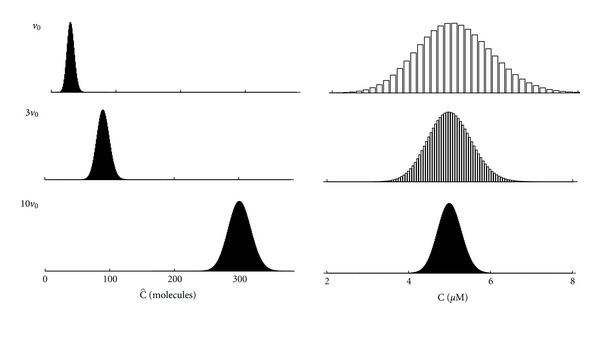
Steady-state probability distribution of the number of calcium ions ($\hat{\text{C}}$, left column) and subspace calcium concentration (C, right column) for subspace volume of *v*_0_ = 10^−17^ liters and subspaces that are 3 and 10 times larger. Parameters: *α* = 0.049 *μ*M/ms, *β* = 0.01 ms^−1^, *c*_*∞*_ = 0.1 *μ*M; the steady-state expected subspace calcium concentration is E[C] = *c*_∗_ = 5 *μ*M.

Figure 2 illustrates fluctuation amplitudes in the minimal subspace model by plotting the steady-state probability distribution of $\hat{\text{C}}$ and C (left and right columns, resp.). In the first row, using subspace volume of *v* = *v*_0_ = 10^−17^ liters and influx rate of *α* = 0.049 *μ*M/ms, the expected calcium concentration is E[C] = *c*_∗_ = *α*/*β* + *c*_*∞*_ = 5 *μ*M, and the expected number of subspace calcium ions is $\text{E}[\hat{\text{C}}]=v_{0}c_{*}=30$. In both cases the coefficient of variation is $1/\sqrt{30}=0.18$ (the spread of the distributions as illustrated is due to the different *x*-axis scales). The following rows of Figure 2 show that in a subspace three or ten times larger (*v* = 3*v*_0_ or 10*v*_0_), the coefficient of variation drops to 0.11 and 0.058, respectively, when the calcium influx rate is scaled to result in the same expected calcium concentration (*c*_∗_ fixed, see (14)). As might be expected, concentration fluctuations in the stochastic model are more pronounced for small volumes and become negligible for large volumes, because $\text{CV}[\text{C}]=1/\sqrt{vc_{*}}\to 0$ as *v* → *∞* for fixed *c*_∗_.

Most importantly, the deterministic and stochastic descriptions of this minimal subspace model agree in the following sense: the expected value of the fluctuating calcium concentration in the stochastic model E[C] = *c*_∗_ = *α*/*β* + *c*_*∞*_ is equal to the steady-state of the deterministic ODE that neglects concentration fluctuations (found by setting the left hand side of (1) to zero). Readers familiar with fluctuations in biochemical models will understand that this agreement is a consequence of the fact that the minimal subspace model involves three elementary reactions, all of which are zeroth or first order (see arrows in Figure 1).

#### 2.1.4. Moment Calculation

The numerical results presented above can be obtained analytically by considering the dynamics of the moments of the number of calcium ions in the subspace, defined as

(15) $$\begin{array}{c} \mu_{q}=\sum_{n=0}^{\infty }n^{q}p_{n}. \end{array}$$

By conservation of probability, the zeroth moment *μ*_0_ = 1 and the first moment is the expected number of calcium ions in the subspace (9),

(16) $$\begin{array}{c} \mu_{1}=\text{E}\left[\hat{\text{C}}\right]. \end{array}$$

The second moment *μ*_2_ is related to the variance of the number of calcium ions via

(17) $$\begin{array}{c} \mathrm{Var}\left[\hat{\text{C}}\right]=\mu_{2}-{\left(\mu_{1}\right)}^{2}. \end{array}$$

By differentiating (15) with respect to time and substituting for the time derivatives using the master equation (5), it can be shown that the zeroth moment is constant (*dμ*_0_/*dt* = 0) and, furthermore,

(18) $$\begin{array}{c} \frac{d\mu_{1}}{dt}=\overset{-}{\alpha }-\beta \mu_{1},\\ \frac{d\mu_{2}}{dt}=\overset{-}{\alpha }+\left(2\overset{-}{\alpha }+\beta \right)\mu_{1}-2\beta \mu_{2}, \end{array}$$

where we have used *μ*_0_ = 1. Setting the left hand sides of these equations to zero, we see that steady-state first and second moment are $\mu_{1}=\overset{-}{\alpha }/\beta$ and $\mu_{2}=\overset{-}{\alpha }/\beta +{\left(\overset{-}{\alpha }/\beta \right)}^{2}$, consistent with (11) and (17).

### 2.2. Stochastic Subspace Model with Calcium Regulation

**Figure 3:**
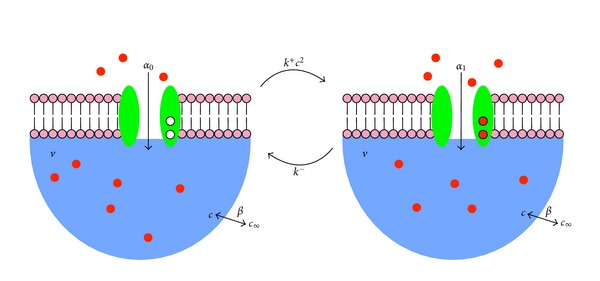
Diagram of the components and fluxes in a subspace model that includes calcium-regulated calcium influx. A single calcium channel (with two calcium binding sites) is associated with a subspace of volume *v*. The calcium influx rate is *α*_0_ and *α*_1_ when calcium is unbound and bound, respectively, and the transition rates between these states are *k* ^+^ *c* ^2^ and *k* ^−^, where *c* is the subspace calcium concentration. Subspace calcium is passively coupled at rate *β* to the bulk cytosol with constant concentration *c*_*∞*_.

 This section augments the subspace model presented above to include calcium regulation of a calcium channel (see Figure 3). We assume that calcium binding instantaneously modifies the conductance of the channel, that is, the rate of calcium influx into the domain is *α*_0_ when the channel is calcium-free and *α*_1_ when the channel is calcium-bound. We further assume the channel has two binding sites for calcium and, for simplicity, approximate rapid sequential binding of calcium ions with instantaneous binding. Thus, the transitions between the two distinct states of the subspace (the so-called “stochastic functional unit" or “calcium release unit") occur at rates *k* ^+^ *c* ^2^ and *k* ^−^, respectively, (Figure 3, curved arrows). Note that the rate constant *k* ^−^ has units of ms^−1^, *k* ^+^ has units of *μ*M^−2^ ms^−1^, and the dissociation constant for calcium binding, denoted by *κ*, has units of *μ*M and is given by *κ* ^2^ = *k* ^−^/*k* ^+^.

#### 2.2.1. Stochastic Model

 Let us denote the states of the stochastic system by (*n*, 0) and (*n*, 1), where *n* ∈ {0,1,…, *∞*} and the second element of the ordered pairs, either 0 or 1, indicates calcium-free and bound channel, respectively. With a little thought we can sketch the following state-transition diagram for the stochastic subspace model with calcium influx,

(19) $$\begin{array}{c} \begin{array}{cccccccccc} \left(0,0\right) & \overset{{\overset{-}{\alpha }}_{0}}{\underset{\beta }{⇌}} & \left(1,0\right) & \overset{{\overset{-}{\alpha }}_{0}}{\underset{2\beta }{⇌}} & \left(2,0\right) & \overset{{\overset{-}{\alpha }}_{0}}{\underset{3\beta }{⇌}} & \left(3,0\right) & \overset{{\overset{-}{\alpha }}_{0}}{\underset{4\beta }{⇌}} & \left(4,0\right) & \cdots \\ & & & & \begin{array}{ccc} 2{\overset{-}{k}}^{+} & ⥯ & k^{-} \end{array} & & \begin{array}{ccc} 6{\overset{-}{k}}^{+} & ⥯ & k^{-} \end{array} & & \begin{array}{ccc} 12{\overset{-}{k}}^{+} & ⥯ & k^{-} \end{array} & \\ & & & & \left(0,1\right) & \overset{{\overset{-}{\alpha }}_{1}}{\underset{\beta }{⇌}} & \left(1,1\right) & \overset{{\overset{-}{\alpha }}_{1}}{\underset{2\beta }{⇌}} & \left(2,1\right) & \cdots \end{array}, \end{array}$$

where ${\overset{-}{k}}^{+}=k^{+}/v^{2}$. The rate of calcium binding to the channel in the stochastic model is inversely proportional to the square of the volume, because of the concentration dependence of the association reaction ($k^{+}c^{2}=k^{+}{\hat{c}}^{2}/v^{2}$). The downward transitions between states (*n*, 0) and (*n* − 2,1) include the combinatorial coefficient, *n*(*n* − 1), double the number of ways that two indistinguishable calcium ions can be chosen from the *n* ions in the subspace. This factor of two is required so that the microscopic propensity ${\overset{-}{k}}^{+}$ agrees with the macroscopic rate *k* ^+^ *c* ^2^ for large *n* and *v* with *c* = *n*/*v* fixed, that is,

(20) $$\begin{array}{c} n\left(n-1\right){\overset{-}{k}}^{+}=n\left(n-1\right)\frac{k^{+}}{v^{2}}=k^{+}\left(c^{2}-\frac{c}{v}\right), \end{array}$$

an expression that approaches *k* ^+^ *c* ^2^ as *v* → *∞* [28].

#### 2.2.2. Master Equation

Let us write *p*_*n*_^0^(*t*) to indicate the probability that at time *t* the channel is calcium-free and $\hat{\text{C}}=n$. Similarly, *p*_*n*_^1^(*t*) is the probability that $\hat{\text{C}}(t)=n$ and the channel is calcium bound. Reading off the transition rates from the state-transition diagram (19), we write the following master equation for the calcium-regulated channel and subspace:

(21) dpn0dt=−[α−0+nβ+n(n−1)k−+]pn0+α−0pn−10+(n+1)βpn+10+k−pn−21,dpn1dt=−[α−1+nβ+k−]pn1+α−1pn−11+(n+1)βpn+11+(n+2)(n+1)k−+pn+20.

Similar to the approach described in the previous section, we define the moments of the number of calcium ions in the subspace *jointly distributed with the state of the channel*, as follows:

(22) $$\begin{array}{c} \mu_{q}^{0/1}=\sum_{n=0}^{\infty }n^{q}p_{n}^{0/1}, \end{array}$$

where the superscript 0/1 indicates either index occurring on both the left and right hand sides of the equality. Note that the zeroth moments sum to unity by conservation of probability (*μ*_0_^0^ + *μ*_0_^1^ = 1). The expected number of calcium ions in the subspace *conditioned on the channel being calcium free or bound*, respectively, is given by

(23) $$\begin{array}{c} {\text{E}}^{0/1}\left[\hat{\text{C}}\right]=\frac{\sum_{n=0}^{\infty }np_{n}^{0/1}}{\sum_{n=0}^{\infty }p_{n}^{0/1}}=\frac{\mu_{1}^{0/1}}{\mu_{0}^{0/1}}. \end{array}$$

Similarly the second moments *μ*_2_^0/1^ are related to the conditional variances via

(24) $$\begin{array}{c} {\mathrm{Var}}^{0/1}\left[\hat{\text{C}}\right]=\frac{\mu_{2}^{0/1}}{\mu_{0}^{0/1}}-{\left(\frac{\mu_{1}^{0/1}}{\mu_{0}^{0/1}}\right)}^{2}. \end{array}$$

#### 2.2.3. Moment Calculation

 By differentiating (22) with respect to time and substituting for the time derivatives using (21), it can be shown that the time-derivatives of the zeroth-moments, *μ*_0_^0^ and *μ*_0_^1^—that is, the probability of the channel being in the calcium free or bound state—are given by

(25) $$\begin{array}{c} \frac{d\mu_{0}^{0}}{dt}=-{\overset{-}{k}}^{+}\mu_{2}^{0}+{\overset{-}{k}}^{+}\mu_{1}^{0}+k^{-}\mu_{0}^{1}, \end{array}$$

(26) $$\begin{array}{c} \frac{d\mu_{0}^{1}}{dt}={\overset{-}{k}}^{+}\mu_{2}^{0}-{\overset{-}{k}}^{+}\mu_{1}^{0}-k^{-}\mu_{0}^{1}, \end{array}$$

where we note that *dμ*_0_^0^/*dt* + *dμ*_0_^1^/*dt* = 0 and *μ*_0_^0^ + *μ*_0_^1^ = 1. In the same way, the equations for the first moments, *μ*_1_^0^ and *μ*_1_^1^, are found to be

(27) $$\begin{array}{c} \frac{d\mu_{1}^{0}}{dt}={\overset{-}{\alpha }}_{0}\mu_{0}^{0}-\beta \mu_{1}^{0}-{\overset{-}{k}}^{+}\mu_{3}^{0}+{\overset{-}{k}}^{+}\mu_{2}^{0}+k^{-}\mu_{1}^{1}+2k^{-}\mu_{0}^{1},\\ \frac{d\mu_{1}^{1}}{dt}={\overset{-}{\alpha }}_{1}\mu_{0}^{1}-\beta \mu_{1}^{1}+{\overset{-}{k}}^{+}\mu_{3}^{0}-3{\overset{-}{k}}^{+}\mu_{2}^{0}+2{\overset{-}{k}}^{+}\mu_{1}^{0}-k^{-}\mu_{1}^{1}. \end{array}$$

Setting the left hand side of (25) to zero, we find that the steady-state probability of a calcium-bound channel is

(28) $$\begin{array}{c} \mu_{0}^{1}=\frac{\mu_{2}^{0}-\mu_{1}^{0}}{\kappa^{2}v^{2}}=\frac{{\text{E}}^{0}\left[{\text{C}}^{2}\right]-{\text{E}}^{0}\left[\text{C}\right]/v}{\kappa^{2}+{\text{E}}^{0}\left[{\text{C}}^{2}\right]-{\text{E}}^{0}\left[\text{C}\right]/v}, \end{array}$$

where in the second equality we have used $\mu_{2}^{0}=\mu_{0}^{0}{\text{E}}^{0}[{\hat{\text{C}}}^{2}]=\mu_{0}^{0}v^{2}{\text{E}}^{0}[{\text{C}}^{2}]$.

Note that as the volume increases (*v* → *∞*), E^0^[C]/*v* becomes negligible compared to E^0^[C^2^], while E^0^[C^2^] → (E^0^[C])^2^ as the conditional variance goes to zero (Var^0^[C] → 0). Thus, in the large system limit, the probability that the channel is in the calcium-bound state is given by

(29) $$\begin{array}{c} \mu_{0}^{1}=\frac{{\left({\text{E}}^{0}[\text{C}]\right)}^{2}}{\kappa^{2}+{\left({\text{E}}^{0}[\text{C}]\right)}^{2}}. \end{array}$$

In the case of a calcium-activated channel, *μ*_0_^1^ is the open probability.

#### 2.2.4. Analysis of Concentration Fluctuations

 The moment analysis in the previous section suggests that the expected calcium concentration in the subspace given by

(30) $$\begin{array}{c} \text{E}\left[\text{C}\right]=\mu_{0}^{0}{\text{E}}^{0}\left[\text{C}\right]+\mu_{0}^{1}{\text{E}}^{1}\left[\text{C}\right] \end{array}$$

and the probability that a calcium-activated channel is open, *p*_open_ = *μ*_0_^1^, may depend on the subspace volume. In order to analyze the effect of small system size and concentration fluctuations at steady-state, we integrated (21) and determined the probability distributions {*p*_*n*_^0/1^} for various model parameters.

**Figure 4:**
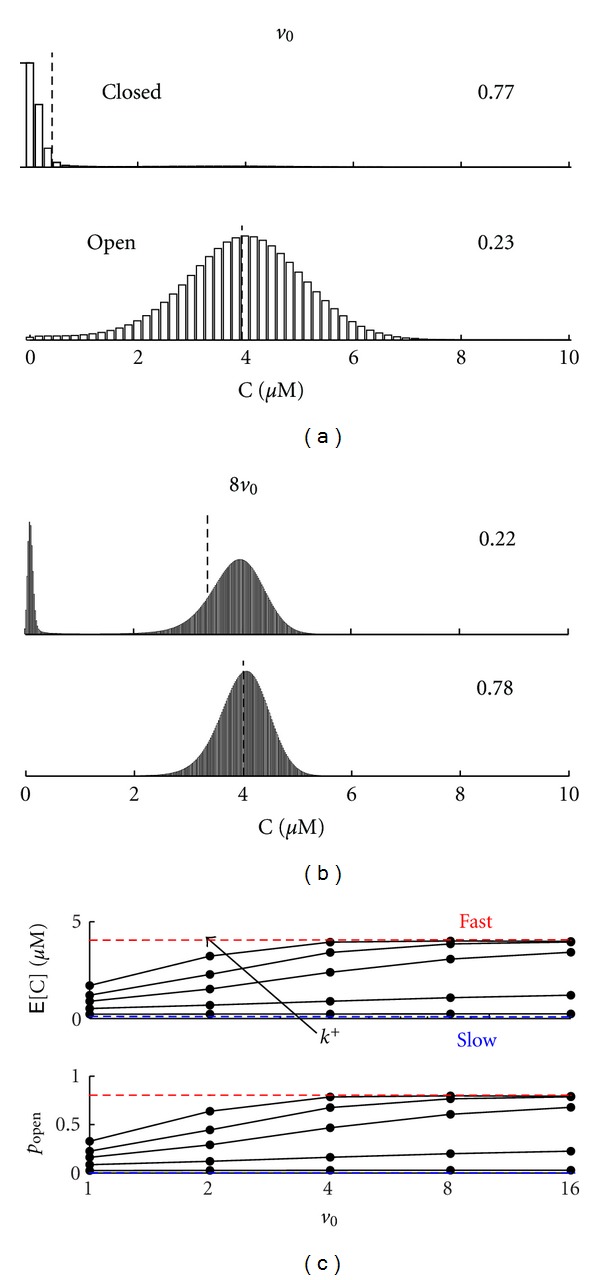
Subspace volume-dependence of calcium fluctuations and open probability of a calcium-activated channel. Steady-state probability distribution for *v* = *v*_0_ (a) and 8*v*_0_ (b) for the calcium-activated channel (*κ* = 2 *μ*M, *k* ^+^ = 0.05 *μ*M^−2^ ms^−1^) [29, 30]. (c) Steady-state E[C] and *p*_open_ = *μ*_0_^1^ for integer multiples of the unitary volume *v*_0_ and different rate constants for calcium binding *k* ^+^ (0.005 to 0.15 *μ*M^−2^ ms^−1^) with *κ* fixed.

Figures 4(a) and 4(b) show the probability distribution for *v* = *v*_0_ and 8*v*_0_ using a representative set of parameters (see caption). In these calculations, the channel is closed when calcium-free and open when calcium-bound, that is,

(31) $$\begin{array}{c} {\overset{-}{\alpha }}_{0}=v\beta c_{\infty }<v\beta c_{*}=v\left(\alpha +\beta c_{\infty }\;\right)={\overset{-}{\alpha }}_{1}. \end{array}$$

For this reason, Figures 4(a) and 4(b) show a conditional expectation for the calcium concentration (vertical dotted lines) that is greater when the channel is calcium bound (E^1^[C] > E^0^[C]). Note that the eight-fold increase in system size leads to a significant increase in the channel open probability, that is, *p*_open_ = *μ*_0_^1^ = 0.23 and 0.78 for *v* = *v*_0_ and 8*v*_0_, respectively. Thus, the open probability of the channel is significantly influenced by the subspace volume, in spite of the fact that the calcium influx rate is scaled so that in the absence of calcium-regulation there is no effect of volume (*α* constant, as in Section 2.1). Comparison of Figures 4(a) and 4(b) also shows a qualitative change in the probability distribution of the subspace calcium concentration (unimodal when *v* = *v*_0_, bimodal when *v* = 8*v*_0_).

Figure 4(c) shows the expected calcium concentration E[C] (30) and open probability (*p*_open_ = *μ*_0_^1^) for the calcium-activated channel as a function of subspace volume *v* and different rate constants for calcium binding *k* ^+^ (fixed dissociation constant *κ*). Both E[C] and *p*_open_ increase with subspace volume *v*, that is, the restricted volume of a physiological subspace leads to an open probability and expected calcium concentration that is less than predicted in the corresponding (approximate) continuous description.

Both the open probability and expected calcium concentration asymptotically approach values in a range that are easily precalculated. For example, E[C] and *p*_open_ must be greater than the values obtained by assuming channel gating is extremely slow, in which case E[C] ≈ *c*_*∞*_ and *p*_open_ ≈ *c*_*∞*_^2^/(*κ* ^2^ + *c*_*∞*_^2^), because the transition from the calcium-free to -bound channel usually occurs with a subspace that is equilibrated with bulk calcium. In addition, E[C] and *p*_open_ are always less than the values obtained under the assumption of rapid channel binding, values given by simultaneous solution of *p*_open_ = *c* ^2^/(*κ* ^2^ + *c* ^2^) and *c* = *p*_open_ *α*/*β* + *c*_*∞*_. These fast and slow system limits are indicated in Figure 4(c) by red and blue horizontal lines, respectively.

**Figure 5:**
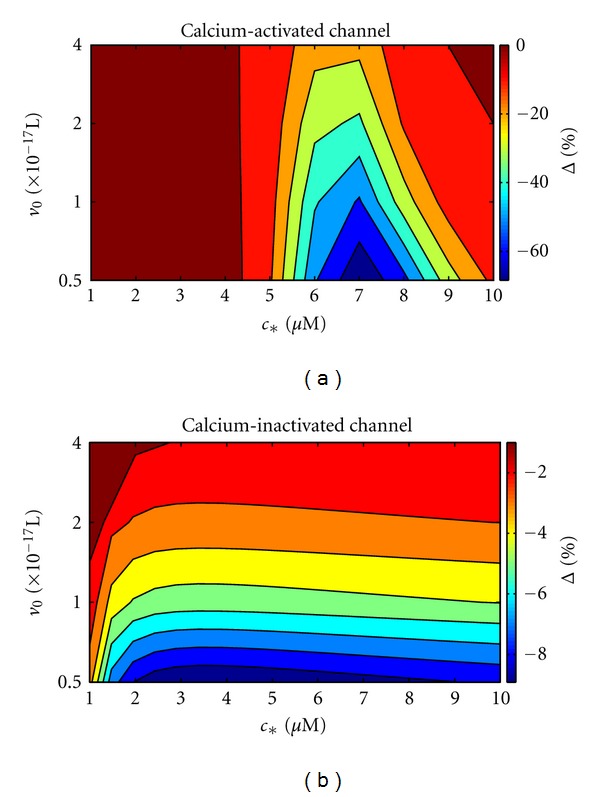
Percentage small system deviation (Δ, (32)) as a function of unitary subspace volume *v*_0_ and influx parameter *c*_∗_ for a single calcium-activated channel (*κ* = 2 *μ*M, *k* ^+^ = 0.005 *μ*M^−2^ ms^−1^) and calcium-inactivated channel (*κ* = 0.63 *μ*M, *k* ^+^ = 0.05 *μ*M^−2^ ms^−1^).

In order to further characterize the effect of subspace volume on the calcium-regulated channel and subspace dynamics, we defined the small system deviation Δ as

(32) $$\begin{array}{c} \Delta =\frac{\text{E}\left[\text{C}\right]-\text{E}{\left[\text{C}\right]}_{\infty }}{\text{E}{\left[\text{C}\right]}_{\infty }}, \end{array}$$

where E[C] is calculated using a system volume of *v* = *v*_0_ and E[C]_*∞*_ is the same quantity calculated in the large system size limit (*v* → *∞*, numerically estimated using *v*⩾10*v*_0_).  Figure 5 shows the small system deviation as a function of unitary subspace volume *v*_0_ and influx parameter *c*_∗_ for a calcium-activated channel. In all cases, Δ was negative, meaning that E[C] for *v* = *v*_0_ was suppressed below the large system size limit and increased with volume (cf. Figure 4(c)). For small *c*_∗_, Δ was near zero. For intermediate values of *c*_∗_ (5–10 *μ*M), the suppression was quite large (Δ ≈ −60%). As *v*_0_ increased, Δ becomes less negative and approaches zero. In general, as *c*_∗_ increased above this range, the suppression ultimately becomes negligible.

For comparison, Figure 5(b) shows the small system deviation for a calcium-inactivated channel. In general, the magnitude of Δ for the calcium-inactivated channel was smaller than the calcium-activated channel. For small *c*_∗_ (1–3 *μ*M), the magnitude of Δ increased with *c*_∗_, while above this range Δ was essentially independent of *c*_∗_. As with the calcium-activated channel, the magnitude of Δ decreased and approached zero as *v*_0_ increased.

### 2.3. Calcium Regulation of Multiple Channels

**Figure 6:**
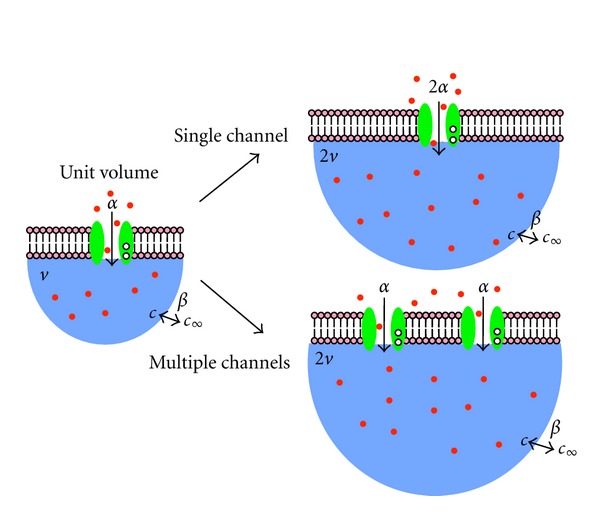
Illustration of two possible volume scalings. For the single channel volume scaling, calcium influx *α* increases proportional to the increase in *v*, but the single channel has only two conductance levels, *α*_0_ and *α*_1_, depending on whether calcium is free or bound. In the alternative scaling, the number of channels increases in proportion to the volume *v*, and when there are many channels the calcium influx rate may take many values between *α*_0_ and *α*_1_.

The previous section analyzed the effect of subspace volume when the influx pathway involves calcium regulation of a single channel. In this section, we assume that the total number of channels increases with subspace volume (see Figure 6). As before, we assume that calcium binding instantaneously modifies the calcium channel conductance, that is, the rate of calcium influx into the domain is determined by *α*_0_ when all channels are calcium-free and *α*_1_ when all channels are calcium-bound.

#### 2.3.1. Deterministic Model

Assuming as before that two free calcium ions C bind to channel B to form the complex C_2_B, we can write the following kinetic scheme:

(33) $$\begin{array}{c} 2\text{C}+\text{B}\overset{k^{+}}{\underset{k^{-}}{⇌}}{\text{C}}_{2}\text{B}. \end{array}$$

The deterministic ODE system that applies in the case of a large subspace volume is

(34) $$\begin{array}{c} \frac{dc}{dt}=\alpha_{0}\frac{b}{b_{t}}+\alpha_{1}\frac{b_{t}-b}{b_{t}}-\beta \left(c-c_{\infty }\right)-k^{+}c^{2}b+k^{-}\left(b_{t}-b\right),\\ \frac{db}{dt}=-k^{+}c^{2}b+k^{-}\left(b_{t}-b\right), \end{array}$$

where we have written *c* = [C] and *b* = [B]. Because the total (calcium-free plus-bound) concentration of channels, *b*_*t*_ = [B]+[C_2_B], is a constant determined by initial conditions, we have eliminated the equation for [C_2_B]. At steady-state the channels will be in equilibrium with subspace calcium, that is, *b*/*b*_*t*_ = *κ* ^2^/*κ* ^2^ + *c* ^2^.Thus, in the case of a calcium-activated channel (*α*_0_ = 0, *α*_1_ > 0), the steady-state calcium concentration satisfies

(35) $$\begin{array}{c} 0=\alpha_{1}\frac{c^{2}}{\kappa^{2}+c^{2}}-\beta \left(c-c_{\infty }\right), \end{array}$$

while in the case of a calcium-inactivated channel (*α*_0_ > 0, *α*_1_ = 0),

(36) $$\begin{array}{c} 0=\alpha_{0}\frac{\kappa^{2}}{\kappa^{2}+c^{2}}-\beta \left(c-c_{\infty }\right). \end{array}$$

**Figure 7:**
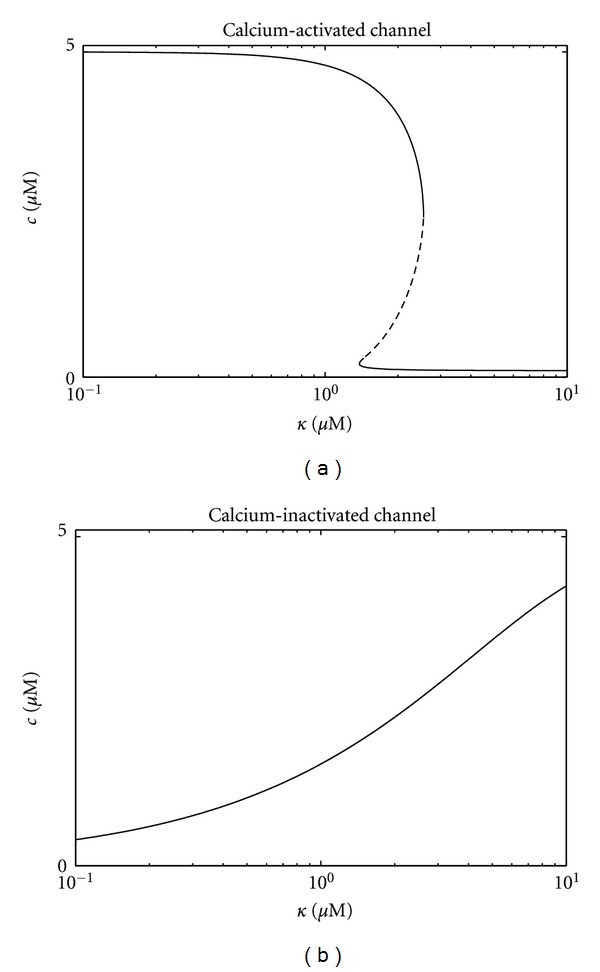
Bifurcation diagram showing the steady-state calcium concentration *c* as a function of dissociation constant *κ* in the deterministic ODE model for a subspace containing multiple calcium-activated (a) and calcium-inactivated (b) channels. Other parameters as in Figure 2.

Figure 7 shows bifurcation diagrams for the steady-state calcium concentration in both cases. For the calcium-activated channel there is a range of *κ* that leads to bistability (Figure 7(a)), while no bistable regime exists for a calcium-inactivated channel (Figure 7(b)).

#### 2.3.2. Stochastic Model

Following the notation developed in the previous section, we write $p_{n}^{m}=\text{P}\{\hat{\text{C}}=n,\hat{{\text{C}}_{2}\text{B}}=m,t\}=\text{P}\{\hat{\text{C}}=n,\hat{\text{B}}={\hat{b}}_{t}-m\}$ for *n* ∈ {0,1,…, *∞*} and $m\in \{0,1,\ldots ,{\hat{b}}_{t}\}$ and, where ${\hat{b}}_{t}$ is the total number of channels (for integer *ℓ*, ${\hat{b}}_{t}=\ell$ when *v* = *ℓv*_0_). The state-transition diagram for the Markov process (not shown) is analogous to (19) but with ${\hat{b}}_{t}+1$ rows as opposed to two. The master equation for the dynamics of the calcium channel and subspace calcium concentration is

(37) dpnmdt=−[α−m+nβ+mk−+n(n−1)(b^t−m)k−+]pnm+α−mpn−1m+(n+1)βpn+1m+(m+1)k−pn−2m+1+(n+2)(n+1)(b^t−m+1)k−+pn+2m−1,

where ${\overset{-}{\alpha }}_{m}=v\left(\alpha_{m}+\beta c_{\infty }\right)$ and

(38) $$\begin{array}{c} \alpha_{m}=\alpha_{0}\frac{{\hat{b}}_{t}-m}{{\hat{b}}_{t}}+\alpha_{1}\frac{m}{{\hat{b}}_{t}}. \end{array}$$

In (38), it is understood that terms in the master equation involving negative indices (i.e., *n* or *m* < 0) evaluate to zero.

#### 2.3.3. Concentration Fluctuations

**Figure 8:**
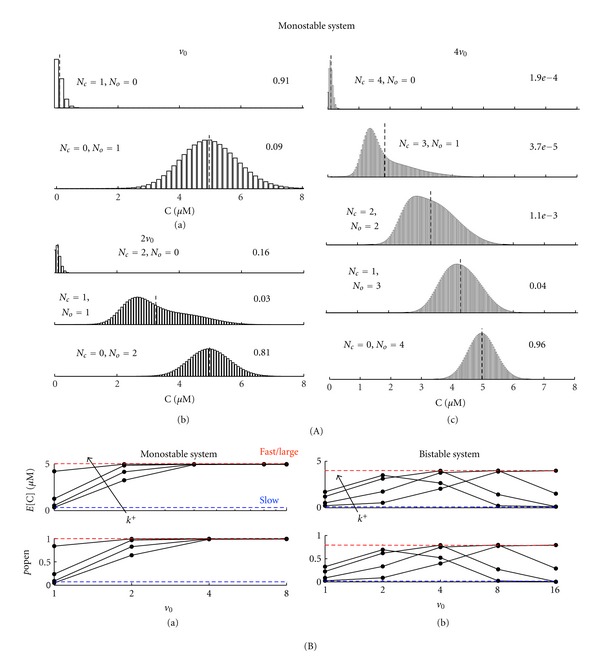
Subspace volume-dependence of concentration fluctuation and channel open probability for multiple calcium-activated channels. (A) Steady-state probability distribution for *v* = *v*_0_ (a), 2*v*_0_ (b), and 4*v*_0_ (c) (*κ* = 0.45 *μ*M, *k* ^+^ = 5 × 10^−4^ *μ*M^−2^ ms^−1^). For each panel, the dashed black line denotes the conditional expected concentration (E^*m*^[C]). The steady-state probability distribution is shown for each possible number of closed (*N*_*C*_) and open (*N*_*O*_) channels. (B) (a) Steady-state E[C] and *p*_open_ for the monostable system as a function of *v* for rate constants of calcium binding (*k* ^+^ = 5 × 10^−5^ to 5 × 10^−3^ *μ*M^−2^ ms^−1^). The fast/large and slow system limits are shown in red and blue, respectively. (b) Steady-state E[C] and *p*_open_ for the bistable system (*κ* = 2 *μ*M) as a function of *v* using *k* ^+^= 0.005 to 0.015 *μ*M^−2^ ms^−1^. In the bistable system, the larger of the two stable equilibrium (large system limit) is shown in red. The smaller equilibrium is approximately equal to the slow system limit (shown in blue).

Figure 8(A) shows the steady-state probability distribution for *v* = *v*_0_, 2*v*_0_ and 4*v*_0_ for a calcium-activated channel with dissociation constant chosen so that the deterministic system is monostable (*κ* = 0.45 *μ*M). For *v* = *v*_0_, there is one channel and two channel states (closed and open). For the closed channel, the distribution of calcium concentration is Poisson-like with conditional mean near *c*_*∞*_, while for the open channel, the conditional mean is near *c*_∗_. For *v* = 2*v*_0_ and 4*v*_0_, there are two or four channels and thus three or five system states, each corresponding to a particular number of free versus bound channels (38). While the conditional expectation of the calcium concentration is always between *c*_*∞*_ and *c*_∗_, these distributions deviate from Poisson.

Figure 8(B)a shows E[C] and *p*_open_ for subspace volumes *v* given by different discrete multiples of the unitary volume *v*_0_. Using parameters that lead to a monostable deterministic ODE system, we find, similar to the case of the single channel (Figure 4), a significant deviation between the expected calcium concentration and open probability for a small subspace as compared to the large system limit (35). As expected, both E[C] and *p*_open_ approached the fast/large system limit as *v* increased. This also occurs for fixed *v* with increasing *k* ^+^, that is, the rate constant for calcium binding. For fixed *κ*, smaller values of *k* ^+^ can cause Δ to approach −100%, that is, the small volume associated with a diadic subspace can almost completely suppress the open probability of a calcium-activated channel. When parameters are chosen so that the deterministic ODE system is bistable, the dependence of E[C] and *p*_open_ is more complex (Figure 8(B)b). Interestingly, the small system deviation in this case is often a biphasic function of system volume.

**Figure 9:**
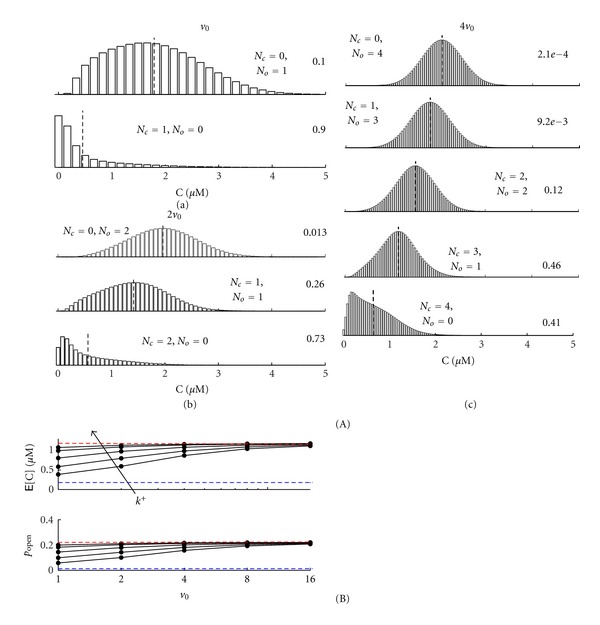
Subspace volume-dependence of concentration fluctuation and channel open probability for multiple calcium-inactivated channels. (A) Steady-state probability distribution for *v* = (a) *v*_0_, (b) 2*v*_0_, and (c) 4*v*_0_ (*κ* = 0.63 *μ*M, *k* ^+^ = 0.005 *μ*M^−2^ ms^−1^). (B) Steady-state E[C] and *p*_open_ as a function of *v* (*k* ^+^ = 0.0015 to 0.15 *μ*M^−2^ ms^−1^).

Figure 9 shows analogous results for calcium-inactivated calcium influx. As with the calcium-activated channel, E[C] and *p*_open_ were suppressed below the fast/large system limit (Figure 9(B)). Δ is often negative, but became negligible as *k* ^+^ increased. Similarly, as *v* increased, both E[C] and *p*_open_ approached the fast/large system limit.

**Figure 10:**
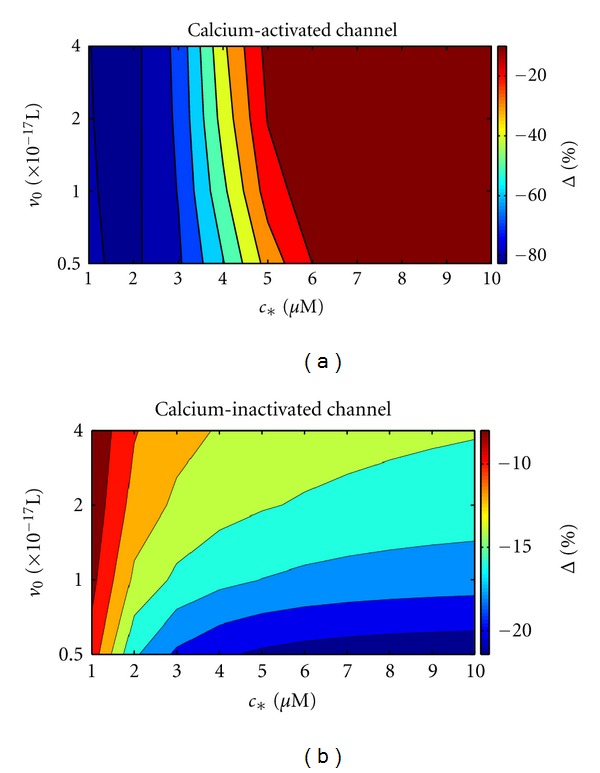
Percentage small system deviation (Δ) as a function of unitary subspace volume *v*_0_ and influx parameter *c*_∗_ for multiple (a) calcium-activated channels (*κ* = 0.45 *μ*M, *k* ^+^ = 0.005 *μ*M^−2^ ms^−1^) and (b) calcium-inactivated channels (*κ* = 0.63 *μ*M, *k* ^+^ = 0.05 *μ*M^−2^ ms^−1^).

Figure 10 summarizes the dependence of the small system deviation (Δ, (32)) on the unitary subspace volume (*v*_0_) and calcium influx parameter (*c*_∗_) for the scaling that involves multiple calcium-activated and -inactivated channels. In all cases, Δ was negative, meaning that E[C] was suppressed compared with the large system values predicted by the deterministic ODE model. Up to 80% suppression was observed for the calcium-activated channel, but for the calcium-inactivated channel the maximum suppression was 20%. In both cases, the largest suppression (most negative Δ) occurs when *p*_open_ is small (i.e., small *c*_∗_ for the calcium-activated channel and large *c*_∗_ for the calcium-inactivated channel). In general, as the unitary volume *v*_0_ is increased, there is less suppression compared to the large system size limit.

### 2.4. The Effect of Domain Size in a Model of Calmodulin-Mediated Channel Regulation

 In the previous sections, we demonstrated that the expected steady-state subspace concentration determined using a minimal model of a calcium-activated or -inactivated channel was volume-dependent and could greatly differ from the steady-state concentration computed from deterministic ODEs. In this section, we show similar results for a state-of-the-art model of calmodulin-mediated calcium regulation.

**Figure 11:**
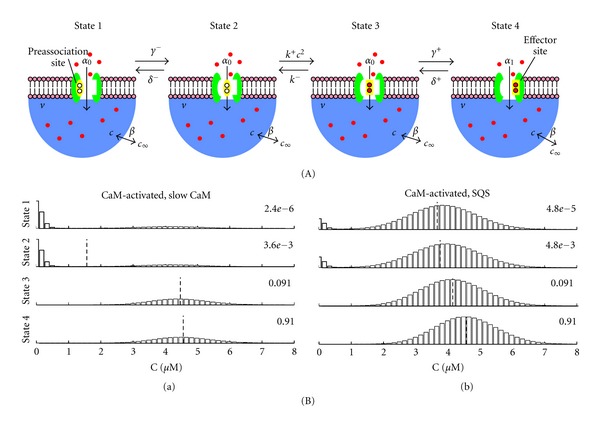
Calmodulin regulation of the calcium channel at steady-state. (A) State diagram of calmodulin regulation of a calcium channel (modified from [31]). (B) Steady-state probability distribution for the calmodulin-activated channel using (a) “slow CaM" and (b) “SQS" parameters [31].

Both the N-lobe and C-lobe of calmodulin have two binding sites for calcium. Depending on the calcium channel type (L, N, or P/Q), calcium binding to the C-lobe has been shown to be responsible for either activation or inactivation of the channel, while N-lobe binding appears to be primarily responsible for channel inactivation [32]. Yue and colleagues demonstrated that the C-lobe responds primarily to the local subspace calcium concentration, while the N-lobe responds to the global or bulk concentration [31]. Tadross et al. developed a 4-state model for calmodulin regulation of the calcium channel (see Figure 11(A)) that includes states for the calmodulin regulator lobe (either the C-lobe or N-lobe) bound to a preassociation site that does not alter channel activity (state 1), unbound (state 2), bound to two calcium ions (state 3), or bound to two calcium ions and an effector site that does alter channel activity (state 4) [31]. Tadross et al. demonstrated that depending on the model parameters, in particular the ratio of the transition rates between states, the calmodulin regulation was sensitive to either local or global calcium levels.

Using this published model as a starting point, we formulated the corresponding discrete Markov model. The elementary reactions for calmodulin-mediated regulation of the channel are

(39) $$\begin{array}{c} S_{1}\overset{\gamma^{-}}{\underset{\delta^{-}}{⇌}}S_{2}\overset{\;k^{+}c^{2}}{\underset{k^{-}}{⇌}}S_{3}\overset{\gamma^{+}}{\underset{\delta^{+}}{⇌}}S_{4}, \end{array}$$

where states *S*_1_ and *S*_2_ are calcium-free, states *S*_3_ and *S*_4_ are calcium-bound, and state *S*_4_ determines the fraction of channels activated (or inactivated) by calmodulin. When it is assumed that a single calmodulin molecule is colocalized with the calcium channel (as in Section 2.2), the master equation takes the following form:

(40) dpn1dt=−(α−0+nβ+γ−)pn1+α−0pn−11+(n+1)βpn+11+δ−pn2,dpn2dt=−[α−0+nβ+δ−+n(n−1)k−+]pn2+α−0pn−12+(n+1)βpn+12+γ−pn1+k−pn−23,dpn3dt=−(α−0+nβ+γ+k−)pn3+α−0pn−13+(n+1)βpn+13+δ+pn4+(n+2)(n+1)k−+pn+22,dpn4dt=−(α−1+nβ+δ+)pn4+α−1pn−14+(n+1)βpn+14+γ+pn3,

where for a calmodulin-activated channel ${\overset{-}{\alpha }}_{0}$ and ${\overset{-}{\alpha }}_{1}$ are given by (31).

Figure 11(B) shows the steady-state probability distribution numerically calculated from these equations. Using a parameter set referred to as “slow CaM,” Tadross et al. showed that calmodulin was primarily sensitive to the local subspace calcium level (representing the C-lobe) when calcium binding to calmodulin was slow [31]. With “slow CaM” parameters, we found that calmodulin bound to the effector site (*S*_4_) had the greatest steady-state probability. Because the calmodulin binding was slow, each conditional distribution had their respective largest peaks near the slow limit (*c*_*∞*_ for states *S*_1_ and *S*_2_, *c*_∗_ for *S*_3_ and *S*_4_) (Figure 11(B)a). Using an alternate parameter set referred to as “SQS," Tadross et al. showed that calmodulin was primarily sensitive to the global calcium level (representing the N-lobe), when calcium binding to calmodulin was fast. Similar to the slow CaM case, state *S*_4_ had the greatest steady-state probability using the SQS parameters. Due to the fast binding kinetics, the conditional distributions were more similar than in the slow CaM case (Figure 11(B)b). For both parameter sets, the calcium concentration distribution for the low occupancy states (*S*_1_ and *S*_2_) were bimodel, with peaks near *c*_*∞*_ and *c*_∗_.

**Figure 12:**
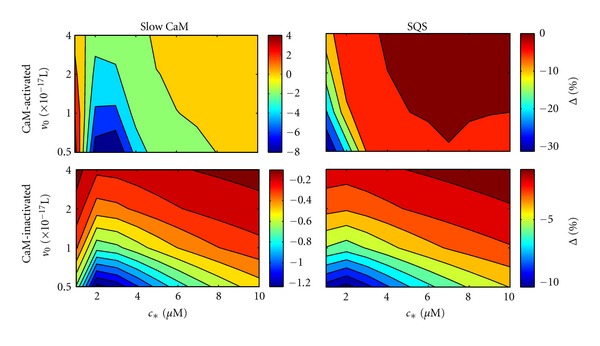
Small system deviation for a single calmodulin-activated and -inactivated channel using “slow CaM” and “SQS” parameters (see text).

Figure 12 shows the small system size suppression Δ for both slow CaM and SQS parameter sets assuming a single calmodulin-regulated channel. As in our simplified model (Figure 5), Δ was quite large in magnitude for some conditions (up to 30% suppression). For the calmodulin-activated channel, the dependence of Δ on *v*_0_ and *c*_∗_ was similar to our simplified model (cf.  Figure 5(a)), decreasing in magnitude and approaching 0 as *v*_0_ or *c*_∗_ increased.

The parameter space for the calmodulin-inactivated channel differed somewhat from our simplified model (Figure 5(b)). For both the slow CaM and SQS parameters, Δ decreased as *c*_∗_ or *v*_0_ increased. Additionally, for both the calmodulin-activated and -inactivated channels, Δ had greater dependence on *v*_0_ for the SQS parameters, which is consistent with calmodulin being more sensitive to the bulk concentration (since increasing *v*_0_ greatly influences the number of ions entering from the bulk).

**Figure 13:**
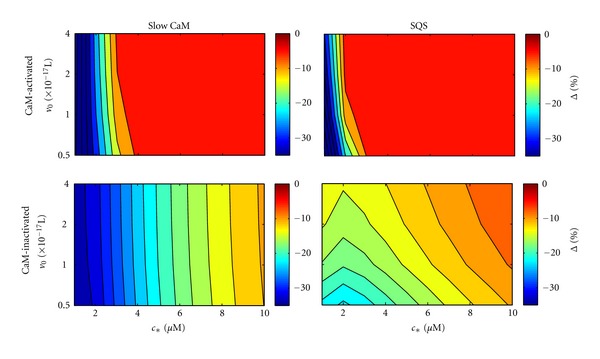
Small system deviation for multiple calmodulin-activated and -inactivated channels, using “slow CaM” and “SQS” parameters.

 We also calculated the small system deviation Δ for the case of multiple calmodulin-regulated channels (Figure 13). For the calmodulin-activated channel, results were similar to our simplified model (Figure 10(a)), in particular Δ approached 0 as both *c*_∗_ and *v*_0_ increased. The magnitude of Δ was smaller for the SQS parameters compared with the slow CaM parameters, consistent with faster kinetics approaching the large system limit and calmodulin being less sensitive to the local calcium concentration. The parameter space for multiple calmodulin-inactivated channels also differed somewhat from our simplified model (Figure 10(b)). In general, the magnitude of Δ decreased as *c*_∗_ increased. However, in contrast with the parameter space using SQS parameters, Δ was fairly insensitive to *v*_0_ using slow CaM parameters, which is consistent with calmodulin being, in this case, less sensitive to the bulk calcium concentration.

## 3. Discussion

We developed a minimal model of a calcium-regulated channel in a small subspace and formulated a Markov model in which each possible discrete state is represented. For small subspace volumes, we found that the value predicted by a continuous-state, deterministic ODE model often deviated from the expected steady-state calcium concentration in the discrete-state, stochastic model. We analyzed how this deviation depends on channel binding kinetics, subspace volume, and calcium influx rate. We demonstrated that the deterministic description also deviated from the stochastic model in a physiologically realistic model of calmodulin-mediated calcium channel regulation.

### 3.1. Physiological Implications

Many studies have modeled the influence of signaling proteins on intracellular and transmembrane ion channel/receptor kinetics, such as calcium/calmodulin-dependent kinase II phosphorylation [33] or beta-adrenergic signaling [34] in cardiac myocytes and glutamate receptor activation in neurons [35]. Many of these signaling interactions occur in small volumes (e.g., the cardiac dyad [4] and neuronal synapse [36]) and include binding interactions with species present in low concentration (calcium and glutamate, resp.). In cardiac myocytes, the local calcium concentration can greatly influence the whole cell response through calcium-induced calcium release, the sodium-calcium exchanger current (which can trigger activation of an action potential), and a host of intracellular signaling pathways [37]. We found that a stochastic model that accounts for the discrete nature of such interactions may deviate from the corresponding deterministic ODE model. Under certain conditions, the small system deviation is negligible, in particular for the case of a large calcium influx rate (Figure 10). During a cardiac action potential, many L-type calcium channels are synchronously opened, and thus the calcium concentration rapidly increases from the micro- to millimolar range. Similarly, following neuronal firing the glutamate concentration in the synaptic cleft can increase several orders of magnitude [35]. In these situations, the deviation of species concentrations from that suggested by deterministic ODE models may not be physiologically significant. However, during resting conditions, the deviation may be significant, and concentration fluctuations due to the small subspace volume could influence channel dynamics (Sections 2.2–2.4). It has been shown that stochastic openings in calcium release channels in the dyadic subspace of cardiac myocytes during diastole can lead to spontaneous calcium release and arrhythmias during heart failure [38]. Our findings demonstrate that a discrete model of the subspace concentration may be important in this physiological context, because it is likely that fluctuations due to the small number of calcium ions play a significant role in generating spontaneous calcium release events.

In addition to demonstrating that a discrete/stochastic model of calcium-regulated calcium influx often deviates from a continuous/deterministic description, we analyzed how subspace volume and concentration fluctuations influence channel dynamics. Because calmodulin effectively colocalizes with the L-type calcium channel [39], the results associated with the “single channel” volume scaling (Figure 5 and Section 2.2) are most relevant. Such colocalization is ubiquitous; many regulators have been shown to colocalize with channels or receptors, including phospholamban with calcium ATPase in the sarcoplasmic reticulum membrane [40], G-protein receptor kinases with G-protein receptors on the cell membrane [41], and Bax with voltage-dependent ion channels in the mitochondrial membrane [42]. Additionally, the volume of diadic subspaces can be greatly altered during pathophysiological conditions. For example, the L-type calcium channels and ryanodine receptors localization in the dyad is disrupted during heart failure and the subspace volume in which these channels reside is much greater in heart failure than during physiological conditions [43]. Our findings show that when a small number of molecules are present in the subspace (small *v*_0_ and *c*_∗_), subspace volume can greatly influence the steady-state properties of stochastically gating channels (Figures 10 and 13).

### 3.2. Relation to Prior Studies

Prior work by our lab has investigated calcium channel regulation through a host of various mechanisms. Groff and Smith investigated the influence of inactivation on calcium spark dynamics in a channel regulated by both calcium-activation and -inactivation [44]. Mazzag et al. demonstrated that residual calcium that accumulates in a subspace during channel openings can influence channel gating [27]. Perhaps more relevant to this study of how concentration fluctuations depend on the subspace volume and influence average rates of calcium binding, channel gating, and calcium influx, Smith and coworkers previously investigated how the number of subspace domains and the number of channels per subspace can influence cellular responses. Williams et al. demonstrated that a population of subspace domains can be represented by a probability density approach and can be utilized to simulate global calcium dynamics [45]. Hartman et al. utilized a model of a small number of coupled calcium activated channels to predict the global calcium release dynamics in response to pharmacological modification of single channel kinetics [46]. However, this study is the first to compare a model of calcium channel regulation accounting for the finite subspace volume (and using a discrete representation of the number of subspace calcium ions) with the corresponding ODE formulation that assumes a large system size (and uses a continuous representation of calcium concentration).

Only a few previous studies have utilized a discrete representation of calcium ions in the context of cardiac myocyte subspace dynamics. Winslow and colleagues simulated the spatial location of discrete diffusing calcium ions, as well as the spatial structure and geometry of the L-type calcium channel and ryanodine receptor in the cardiac dyad [47]. They demonstrate that stochastic fluctuations produce variability in the L-type calcium channel-ryanodine receptor signaling interactions (specifically excitation-contraction coupling gain), but their analysis does not distinguish between the influence of fluctuations due to channel gating, calcium diffusion, and small calcium ion number. Similar to this study, von Wegner and Fink presented a stochastic model of the L-type calcium channel, incorporating calcium diffusion, buffering, and channel gating and conductance, and they demonstrated how calcium concentration fluctuations could influence downstream signalling pathways [48]. Our results are novel in their focus on the influence of subspace volume and the kinetics of calcium-regulation of an L-type channel. Most importantly, we provide a thorough analysis of the deviation of the approximate deterministic description from the full stochastic model and clarify the conditions leading to large versus small deviations.

Previous studies have modeled biochemical reaction networks using master equations and compared results with deterministic ODE models. McQuarrie demonstrated in 1963 that for first-order reactions, the expected steady-state concentrations derived from the chemical master equation and deterministic ODEs agree [5]. In Section 2.1, calcium influx from an unregulated channel is modeled using zeroth- and first-order reactions and, consequently, the stochastic and deterministic descriptions must agree. Our observation that concentration fluctuations increased as the subspace volume became smaller is consistent with well-understood principles of statistical physics and should come as no surprise [23].

Darvey et al. demonstrated for several generic second-order reactions, the expected concentration computed from the chemical master equation may deviate from the corresponding ODE model [20]. The deviation is typically negative (i.e., Δ < 0), with greatest suppression when concentration fluctuations are large. Other recent studies have demonstrated that the concentrations of species in stochastic biochemical networks can deviate from deterministic ODE descriptions. In agreement with our findings, the deviation is often negative [18, 21, 28], although positive deviation was observed in some biochemical systems [12, 49]. Our findings are consistent with Darvey et al., in that Δ had the greatest magnitude when either the subspace volume or calcium influx rate was small (Figure 10) (both result in larger concentration fluctuations, see (14)).

We found that the small system size deviation was particularly complex in cases where the deterministic ODE, that is, the model appropriate for the large system size limit, is bistable (Figure 8). Lestas et al. recently investigated bistability/bimodality in a network of gene regulation and demonstrated that bistability in the deterministic ODE model did not imply bimodality in the discrete system and, conversely, bimodality in the discrete system did not imply bistability in the corresponding ODEs [50]. We obtained similar results, as bimodality in distribution was not present in the bistable system for *v* = *v*_0_ (Figure 4(a)) but was present for *v* = 8*v*_0_ (Figure 4(b)). Conversely, for the calmodulin-regulated channel, bimodality was present in the distribution for the monostable system (Figure 11). Interestingly, for the bistable system, E[C] computed from the discrete model need not be well approximated by either of the two stable equilibria in the deterministic model; rather, E[C] is given by an intermediate value and can have a complex dependence of subspace volume (Figure 8(C)). But it is important to note that the small system size deviation does not require a bistable deterministic model. The deviation can be quite pronounced even in a monostable deterministic model (Figures 8(B), 9, and 10).

### 3.3. Limitations

 The two-state kinetic models of the calcium channel introduced in Section 2.1 is minimal and should be interpreted as phenomenological (as opposed to statistical) model of single channel kinetics, that is, the topology and parameters of this model were not obtained by fitting to patch clamp recordings [51]. On the other hand, the kinetic model for regulation of the calcium channel presented in Section 2.4 is state-of-the-art. Both minimal and physiologically realistic channel models are affected by the decision to account for (or neglect) fluctuations in calcium concentration that result from the small number of ions in the subspace.

The most significant limitation in the model formulation is our neglect of spatial dynamics of calcium diffusion within the dyadic subspace and the details of the spatial arrangement of the ryanodine receptors [47, 52]. However, for the purposes of the present study, that is, investigation of the influence of concentration fluctuations on the regulation of calcium influx, a nonspatial Markov chain model that includes subspace volume as a model parameter and accounts for the finite number of calcium ions in the domain is sufficient.

Another limitation of the present work is that we focus on *stationary* statistics, for example, the expected value of the steady-state subspace calcium concentration, in our analysis of the deviation of continuous ODE description from the discrete stochastic formulation. Future studies could address how *transient* dynamics, for example, the cellular response to a depolarizing voltage step, excitation-contraction coupling gain, and so forth, are affected by calcium concentration fluctuations resulting from small subspace volume.

## 4. Conclusions

 Our findings demonstrate the physiological relevance of concentration fluctuations in both minimal and realistic models of a calcium-regulated channels associated with subspaces of small volume. The take home message is: *concentration fluctuations do not “average out” in a manner that causes stochastic and deterministic descriptions of subspace dynamics to be equivalent*. Future studies will investigate how subspace calcium concentration fluctuations may influence global calcium dynamics and plasma membrane electrical activity in physiological and pathophysiological conditions.

## References

[1] Kamp TJ, Hell JW (2000). Regulation of cardiac L-type calcium channels by protein kinase A and protein kinase C. *Circulation Research*.

[2] Rose WC, Balke CW, Wier WG, Marban E (1992). Macroscopic and unitary properties of physiological ion flux through L-type Ca2+ channels in guinea-pig heart cells. *Journal of Physiology*.

[3] Greenstein JL, Winslow RL (2002). An integrative model of the cardiac ventricular myocyte incorporating local control of Ca2+ release. *Biophysical Journal*.

[4] Iyer V, Mazhari R, Winslow RL (2004). A computational model of the human left-ventricular epicardial myocyte. *Biophysical Journal*.

[5] McQuarrie DA (1963). Kinetics of small systems. I. *The Journal of Chemical Physics*.

[6] Liang J, Qian H (2009). Computational cellular dynamics based on the chemical master equation: a challenge for understanding complexity. *Journal of Computer Science and Technology*.

[7] Qian H (2011). Nonlinear stochastic dynamics of mesoscopic homogeneous biochemical reaction systems—an analytical theory. *Nonlinearity*.

[8] Qian H, Bishop LM (2010). The chemical master equation approach to nonequilibrium Steady-state of open biochemical systems: linear single-molecule enzyme kinetics and nonlinear biochemical reaction networks. *International Journal of Molecular Sciences*.

[9] Gadgil CJ (2009). Size-independent differences between the mean of discrete stochastic systems and the corresponding continuous deterministic systems. *Bulletin of Mathematical Biology*.

[10] Arslan E, Laurenzi IJ (2008). Kinetics of autocatalysis in small systems. *Journal of Chemical Physics*.

[11] Higham DJ (2008). Modeling and simulating chemical reactions. *SIAM Review*.

[12] Gómez-Uribe CA, Verghese GC (2007). Mass fluctuation kinetics: capturing stochastic effects in systems of chemical reactions through coupled mean-variance computations. *Journal of Chemical Physics*.

[13] Calder M, Duguid A, Gilmore S, Hillston J (2006). Stronger computational modelling of signalling pathways using both continuous and discrete-state methods. *Computational Methods in Systems Biology*.

[14] Wolkenhauer O, Ullah M, Kolch W, Cho KH (2004). Modeling and simulation of intracellular dynamics: choosing an appropriate framework. *IEEE Transactions on Nanobioscience*.

[15] Turner TE, Schnell S, Burrage K (2004). Stochastic approaches for modelling in vivo reactions. *Computational Biology and Chemistry*.

[16] Vasudeva K, Bhalla US (2004). Adaptive stochastic-deterministic chemical kinetic simulations. *Bioinformatics*.

[17] Qian H, Saffarian S, Elson EL (2002). Concentration fluctuations in a mesoscopic oscillating chemical reaction system. *Proceedings of the National Academy of Sciences of the United States of America*.

[18] Laurenzi IJ (2000). An analytical solution of the stochastic master equation for reversible bimolecular reaction kinetics. *Journal of Chemical Physics*.

[19] Wang H, Li Q (1998). Master equation analysis of deterministic chemical chaos. *Journal of Chemical Physics*.

[20] Darvey IG, Ninham BW, Staff PJ (1966). Stochastic models for second-order chemical reaction kinetics. The equilibrium state. *The Journal of Chemical Physics*.

[21] Zheng Q, Ross J (1991). Comparison of deterministic and stochastic kinetics for nonlinear systems. *The Journal of Chemical Physics*.

[22] Goutsias J (2007). Classical versus stochastic kinetics modeling of biochemical reaction systems. *Biophysical Journal*.

[23] Keizer JE (1987). *Statistical Thermodynamics of Nonequilibrium Processes*.

[24] Wray S, Burdyga T (2010). Sarcoplasmic reticulum function in smooth muscle. *Physiological Reviews*.

[25] Winslow RL, Greenstein JL (2011). Cardiac myocytes and local signaling in nano-domains. *Progress in Biophysics and Molecular Biology*.

[26] Cannell MB, Kong CHT (2011). Local control in cardiac E-C coupling. *Journal of Molecular and Cellular Cardiology*.

[27] Mazzag B, Tignanelli CJ, Smith GD (2005). The effect of residual Ca2+ on the stochastic gating of Ca 2+-regulated Ca2+ channel models. *Journal of Theoretical Biology*.

[28] Wu J, Vidakovic B, Voit EO (2011). Constructing stochastic models from deterministic process equations by propensity adjustment. *BMC Systems Biology*.

[29] Györke I, Györke S (1998). Regulation of the cardiac ryanodine receptor channel by luminal Ca2+ involves luminal Ca2+ sensing sites. *Biophysical Journal*.

[30] Huertas MA, Smith GD (2007). The dynamics of luminal depletion and the stochastic gating of Ca2 +-activated Ca2 + channels and release sites. *Journal of Theoretical Biology*.

[31] Tadross MR, Dick IE, Yue DT (2008). Mechanism of local and global Ca2+ sensing by calmodulin in complex with a Ca2+ channel. *Cell*.

[32] Liang H, DeMaria CD, Erickson MG, Mori MX, Alseikhan BA, Yue DT (2003). Unified mechanisms of Ca2+ regulation across the Ca 2+ channel family. *Neuron*.

[33] Hashambhoy YL, Greenstein JL, Winslow RL (2010). Role of CaMKII in RyR leak, EC coupling and action potential duration: a computational model. *Journal of Molecular and Cellular Cardiology*.

[34] Heijman J, Volders PGA, Westra RL, Rudy Y (2011). Local control of *β*-adrenergic stimulation: effects on ventricular myocyte electrophysiology and Ca2+-transient. *Journal of Molecular and Cellular Cardiology*.

[35] Pendyam S, Mohan A, Kalivas PW, Nair SS (2009). Computational model of extracellular glutamate in the nucleus accumbens incorporates neuroadaptations by chronic cocaine. *Neuroscience*.

[36] Rusakov DA, Min MY, Skibo GG, Savchenko LP, Stewart MG, Kullmann DM (1999). Role of the synaptic microenvironment in functional modification of synaptic transmission. *Neurophysiology*.

[37] Bers DM (2002). Cardiac excitation-contraction coupling. *Nature*.

[38] Chen W, Aistrup G, Andrew Wasserstrom J, Shiferaw Y (2011). A mathematical model of spontaneous calcium release in cardiac myocytes. *American Journal of Physiology*.

[39] Mori MX, Erickson MG, Yue DT (2004). Functional stoichiometry and local enrichment of calmodulin interacting with Ca2+ channels. *Science*.

[40] Stenoien DL, Knyushko TV, Londono MP (2007). Cellular trafficking of phospholamban and formation of functional sarcoplasmic reticulum during myocyte differentiation. *American Journal of Physiology*.

[41] Krupnick J, Benovic J (1998). The role of receptor kinases and arrestins in G protein-coupled receptor regulation. *Annual Review of Pharmacology and Toxicology*.

[42] Narita M, Shimizu S, Ito T (1998). Bax interacts with the permeability transition pore to induce permeability transition and cytochrome c release in isolated mitochondria. *Proceedings of the National Academy of Sciences of the United States of America*.

[43] Song LS, Sobie EA, McCulle S, Lederer WJ, Balke CW, Cheng H (2006). Orphaned ryanodine receptors in the failing heart. *Proceedings of the National Academy of Sciences of the United States of America*.

[44] Groff JR, Smith GD (2008). Calcium-dependent inactivation and the dynamics of calcium puffs and sparks. *Journal of Theoretical Biology*.

[45] Williams GSB, Huertas MA, Sobie EA, Jafri MS, Smith GD (2007). A probability density approach to modeling local control of calcium-induced calcium release in cardiac myocytes. *Biophysical Journal*.

[46] Hartman JM, Sobie EA, Smith GD (2010). Spontaneous Ca2+ sparks and Ca2+ homeostasis in a minimal model of permeabilized ventricular myocytes. *American Journal of Physiology*.

[47] Winslow RL, Tanskanen A, Chen M, Greenstein JL (2006). Multiscale modeling of calcium signaling in the cardiac dyad. *Annals of the New York Academy of Sciences*.

[48] Von Wegner F, Fink RHA (2010). Stochastic simulation of calcium microdomains in the vicinity of an L-type calcium channel. *European Biophysics Journal*.

[49] Grima R (2012). Study of the accuracy of moment-closure approximations for stochastic chemical kinetics. *The Journal of Chemical Physics*.

[50] Lestas I, Paulsson J, Ross NE, Vinnicombe G (2008). Noise in gene regulatory networks. *IEEE Transactions on Automatic Control*.

[51] Sakmann B, Neher E (1995). *Single-Channel Recording*.

[52] Groff JR, Smith GD (2008). Ryanodine receptor allosteric coupling and the dynamics of calcium sparks. *Biophysical Journal*.

